# A network pharmacology study on analgesic mechanism of Yuanhu-Baizhi herb pair

**DOI:** 10.1186/s12906-020-03078-0

**Published:** 2020-09-18

**Authors:** Bobin Mi, Qiushi Li, Tong Li, Jessica Marshall, Jiayang Sai

**Affiliations:** 1 Department of Orthopaedics, Union Hospital, Tongji Medical College, Huazhong University of Science and Technology, Wuhan, 430022 China; 2 Department of Cardiology, Beijing Chaoyang Integrative Medicine Emergency Medical Center, Beijing, 100029 China; 3 Department of oncology, The Third Affiliated Hospital, Beijing University of Chinese Medicine, Beijing, 100029 China; 4 Department of Surgery, Brigham and Women’s Hospital, Harvard Medical School, Boston, 02115, Boston, MA 02115 USA

**Keywords:** Rhizoma Corydalis, Radix angelicae dahuricae, Pain, Analgesic, Network pharmacology

## Abstract

**Background:**

Millions of people are suffering from chronic pain conditions, such as headache, arthritis, cancer. Apart from western medicines, traditional Chinese medicines are also well accepted for pain management, especially in Asian countries. Yuanhu-Baizhi herb pair (YB) is a typical herb pair applied to the treatment of stomach pain, hypochondriac pain, headache, and dysmenorrhea, due to its effects on analgesia and sedation. This study is to identify potentially active compounds and the underlying mechanisms of YB in the treatment of pain.

**Methods:**

Compounds in YB were collected from 3 online databases and then screened by bioavailability and drug likeness parameters. Swiss target prediction was applied to obtain targets information of the active compounds. Pain-related genes were conducted for Gene ontology (GO) and Kyoto Encyclopedia of Genes and Genomes (KEGG) analysis. Protein-protein interaction (PPI) networks of the genes were constructed using Cytoscape software. In addition, the hub genes were screened using maximal clique centrality (MCC) algorithm.

**Results:**

In total, 31 compounds from Yuanhu were screened out with 35 putative target genes, while 26 compounds in Baizhi with 43 target genes were discovered. Hence, 78 potential target genes of YB were selected for further study. After overlap analysis of the 78 genes of YB and 2408 pain-associated genes, we finally achieved 34 YB-pain target genes, as well as 10 hub genes and 23 core compounds. Go enrichment and KEGG pathway analysis indicated that YB had a strong integration with neuro system, which might significantly contribute to antinociceptive effect.

**Conclusion:**

Our data provide deep understanding of the pharmacological mechanisms of YB in attenuating pain. The discovery shed new light on the development of active compounds of YB for the treatment of pain.

## Background

Pain is a common syndrome related to various diseases, such as cancer, fracture, etc. It is estimated that 20% of individuals around the worldwide have some degree of chronic pain [1]. Adequate pain assessment and rational management are essential to improve the quality of life in this population. Although stepwise escalation of analgesic therapy (paracetamol, non-steroidal anti-inflammatory drugs, mild to strong opioids) according to the World Health Organization’s three-step pain ladder works well for majority patients suffering from pain [2], the prevailing adverse effect still exist among patients after using the primary drugs. Thus, adjuvants are always recommended for the management of nociceptive pain to maximize nonopioids and minimize long-term opioid use in patients who may live for decades with a chronic pain syndrome.

Nowadays, accumulative studies provide substantial evidence that traditional Chinese medical therapy, including herbs, formulas, etc. has an additive effect when used in combination with opioids or may be used as single agent treatment for pain relief [3, 4].Yuanhu-Baizhi herb pair (YB), consisting of *Corydalis yanhusuo* W. T. Wang (Yuanhu in Chinese) and *Angelica dahurica* (Fisch.ex Hoffm.) Benth. et Hook. f. (Baizhi in Chinese), is well-known for its analgesic effect [5]. Diverse forms of YB, such as capsules, pills, and oral solution, have been developed and widely used in the treatment of stomachache, headache and dysmenorrhea. In addition, previous studies have proved pain alleviating effects of YB [5–7]. Considering the widely clinical use of Yuanhu and Baizhi on pain management, the active compounds and potential targets of YB on analgesia has yet to be discovered.

Traditional Chinese medicine (TCM) network pharmacology is a preferred method to study herb-compounds-diseases-targets because of its capacity of describing complex interactions between drugs and biological systems and the “multi-component, multi-target, and multi-pathway” characteristics of TCM. Therefore, in the present study, we are committed to screen the active compounds in YB that may modulate pain-related genes. Besides, the underlying mechanism of YB-induced pain relief was investigated.

## Methods

### Active compounds and targets

All of the chemical monomer compounds in YB were retrieved from The Encyclopedia of Traditional Chinese Medicine (ETCM, http://www.nrc.ac.cn:9090/ETCM/), Traditional Chinese Medicine Information Database (TCMID, http://bidd.nus.edu.sg/group/TCMsite/), and TCMGeneDIT (http://tcm.lifescience.ntu.edu.tw/). ETCM includes comprehensive information for the commonly used herbs and formulas of TCM, as well as their ingredients. TCMID is a systemic platform designed to identify informative materials on all aspects of TCM including formulation, herbal composition, chemical composition, etc. TCMGeneDIT serves as a database system providing association information about TCMs, genes, diseases, TCM effects and TCM ingredients automatically mined from vast amount of biomedical literature.

### Pharmacokinetic predictions

Canonical Simplified Molecular-Input Line-Entry System (SMILES) of compounds were collected from three databases, including Swiss ADME (http://www.swissadme.ch/), Pubchem (https://pubchem.ncbi.nlm.nih.gov/), and ChEMBL (https://www.ebi.ac.uk/chembl/). Then five important pharmacology-related properties were obtained from Swiss ADME by accurate searching canonical SMILES, including MW, ALogP, Hbond donor count, and Hbond acceptor count, Rotation bond count. Those properties were applied to the drug likeness evaluation based on Lipinski’s rule of five (RO5) [8]. Systemic evaluation of ADME (absorption, distribution, metabolism, excretion, toxicity) was carried out by ADMETlab (http://admet.scbdd.com/), among which, F (30% Bioavailability) and drug likeness (DL) were collected for further screening. Then, compounds with F (30%) ≥ 30% and DL ≥30% were identified as active compounds.

### Targets prediction

The potential targets of active compounds of YB are predicted by Swiss ADME and collected using a probability ≥30%. The pain-associated target genes were obtained from six databases, including DISGeNET [9], drugbank [10], GeneCards [11], The Online Mendelian Inheritance in Man database (OMIM) [12], Therapeutic Target Database (TTD) [13], and The Human Protein Atlas (THPA) [14]. The species was set to *Homo sapiens*. Venn diagram was drawn for overlap analysis to obtain potential pain-associated target genes of active compounds.

### Network construction

Protein-Protein Interaction (PPI) data were obtained from the Search Tool for the Retrieval of Interacting Genes (STRING) database and a confidence score of > 0.4 was selected to construct PPI network [15]. The networks were generated using Cytoscape (version 3.7.1) to further illustrate scientific interpretation of the complicated relationships among genes.

### Enrichment analysis

Gene Ontology (GO) Enrichment and Kyoto Encyclopedia of Genes and Genomes (KEGG) Pathway Enrichment Analysis were carried out using DAVID tool (https://david.ncifcrf.gov/) [16].

## Results

### Drug likeness and bioavailability analysis for compounds in YB

In this study, we obtained 47 compounds in Yuanhu (*Corydalis yanhusuo* W. T. Wang) and 84 compounds in Baizhi (*Angelica dahurica* (Fisch.ex Hoffm.) Benth. et Hook. f., Supplementary Excel.1). Then we selected compounds using descriptors retrieved from Swiss ADME, results in 43 compounds out of 47 in Yuanhu and 77 out of 84 in Baizhi, respectively. The statistic results of their drug-like property descriptors were listed in Table 1. Further screening was carried out based on F (30%) ≥ 30% and DL ≥ 30% collected from ADMETlab. Finally, we obtained 41 compounds in Yuanhu and 64 compounds in Baizhi (Supplementary Excel. 2).

**Table 1:** Drug-like property descriptors of compounds in YB

Herbs	Descriptors	Median	Mean	Std
Yanhu	MW	341.40	332.74	53.60
	a_acc	5.00	4.86	0.95
	a_don	1.00	0.67	0.84
	logP(o/w)	2.79	2.59	0.88
Baizhi	MW	204.35	208.61	73.31
	a_acc	2.00	2.44	2.30
	a_don	0.00	0.66	0.95
	logP(o/w)	3.24	3.00	1.62

### YB compound-target network

**Fig. 1:**
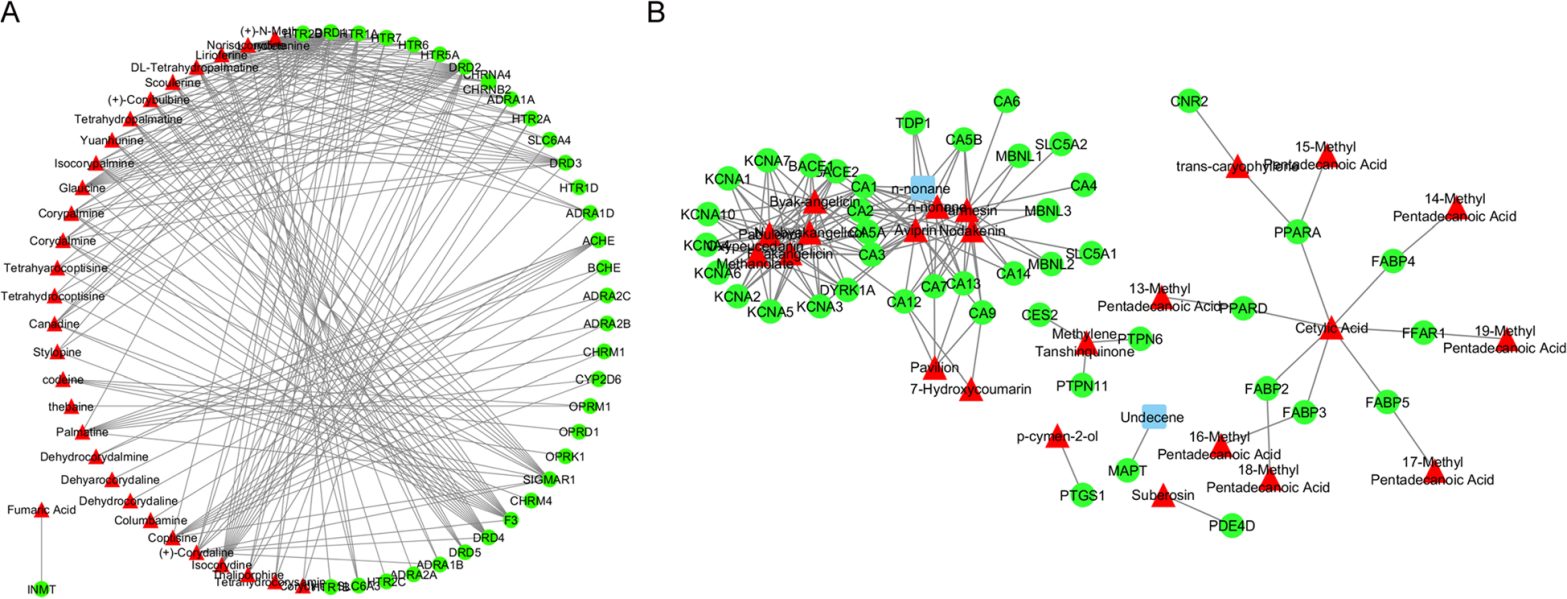
Yuanhu and Baizhi compound–target network. **a** Yuanhu compound–target network. **b** Bai zhi compound–target network. Red represents compounds and green represents the targets of compounds

Swiss Target Prediction was applied to identify target genes of active compounds. After screening using a probability ≥30%, 31 active compounds from Yuanhu with 35 putative target genes, and 26 active compounds in Baizhi with 43 putative target genes were identified in the results. The interaction between active compounds and genes were constructed by Cytoscape. (Fig. 1a, b).

As shown in Fig. 1, the network showed that compounds which connected to the most target genes were tetrahydropalmatine, codeine, lirioferine etc., indicating that these compounds might play a major role in Yuanhu, while cetylic acid, Neobyakangelicol etc., might be the critical compounds in Baizhi. In addition, we listed the compound–candidate target network parameters in Table 2.

**Table 2:** YB compound–candidate target network parameters

Herbs	Network parameter	Values
Yanhu	Number of nodes	64
	Network heterogeneity	0.830
	Average number of neighbors	5.969
	Characteristic path length	3.088
	Shortest paths	3784 (93%)
	Network centralization	0.246
Baizhi	Number of nodes	68
	Network heterogeneity	0.931
	Average number of neighbors	4.824
	Characteristic path length	2.473
	Shortest paths	1930 (42%)
	Network centralization	0.156

### Overlap analysis between YB targets and pain associated targets

**Fig. 2:**
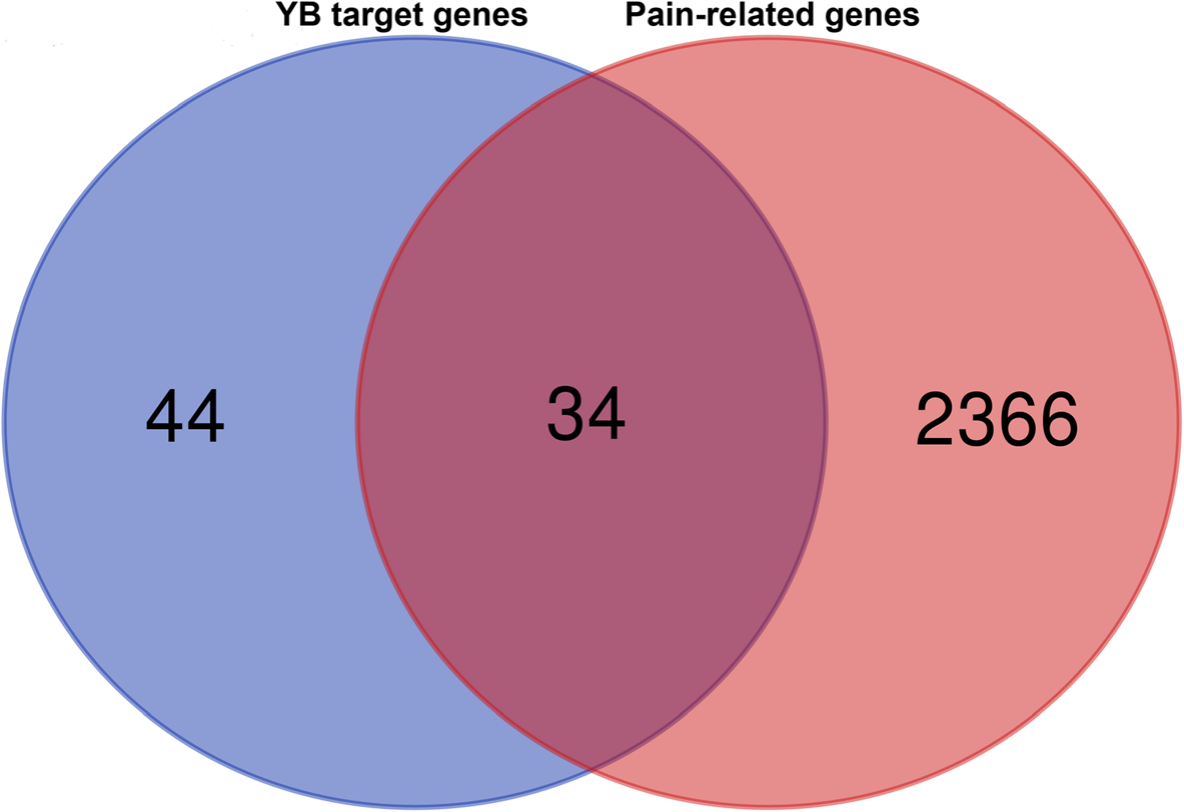
Venn Diagram for the overlap analysis of YB tageted genes and pain related genes

**Fig. 3:**
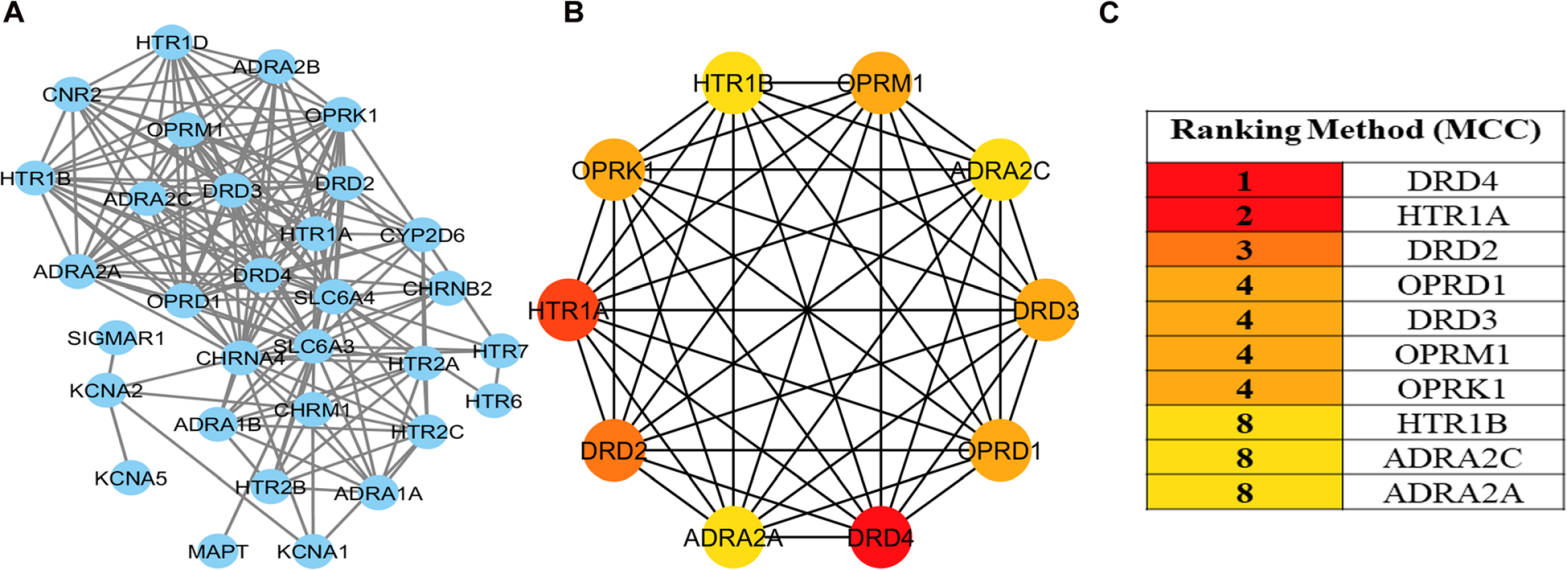
Protein–protein interaction (PPI) network of YB compound targets against pain. **a** The PPI Network constructed by Cytoscape; **b** Hub genes cluster generated from **a**; **c** Hub genes list for YB on pain by MCC method

**Fig. 4:**
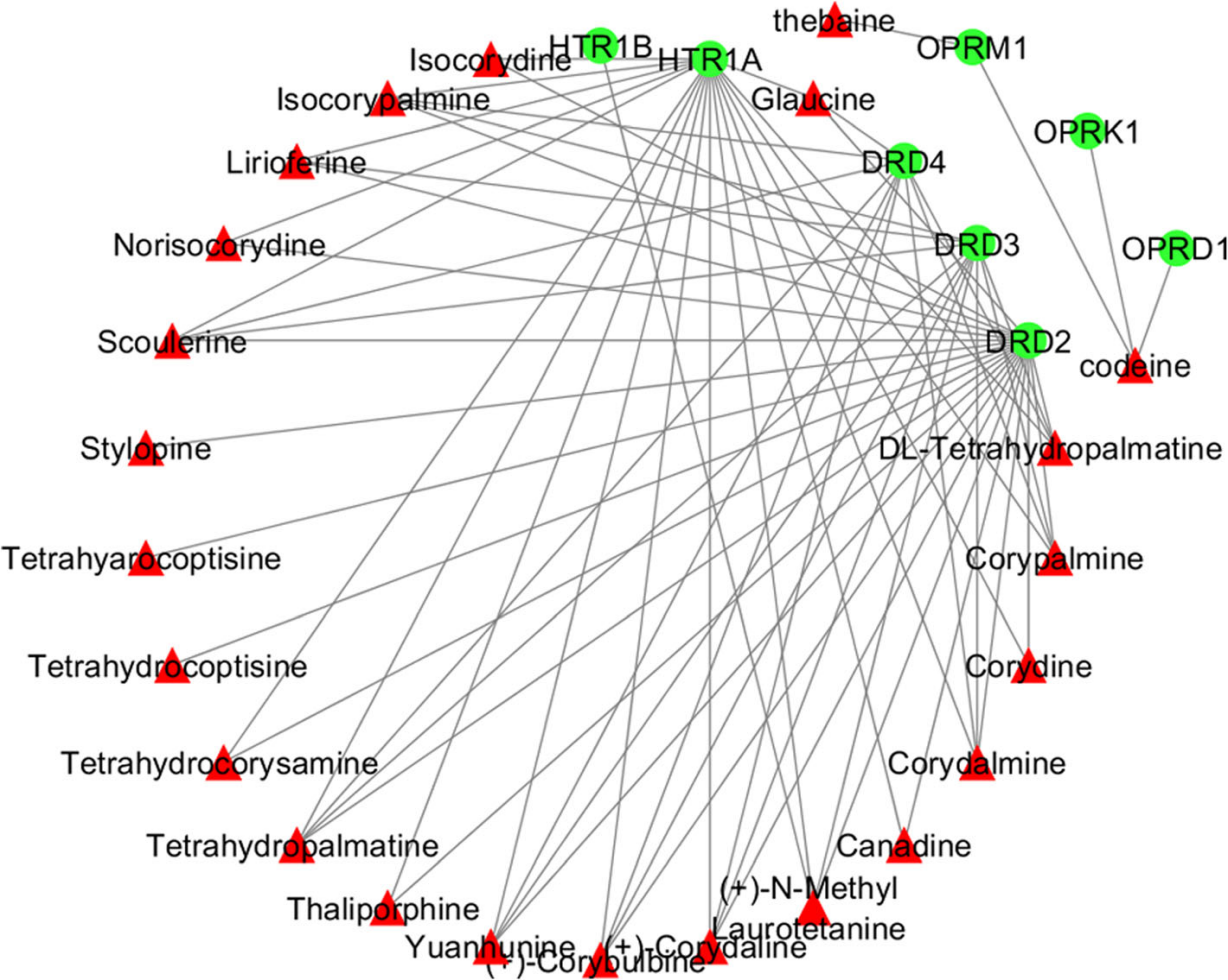
The network for 8 YB-related pain target genes and their interactive YB compounds. Green nodes represent pain-related targeted genes, and red nodes represent YB active compounds

Six databases were mined including TTD, DISGeNET, DrugBank, Genecards, OMIM, and The Human Protein Atlas to screen the pain-related genes. In total, 2408 pain-related genes were obtained. After overlapping analysis, 34 frequently affected therapeutic target genes for pain in active compounds of YB were discovered (Fig. 2). We also created a PPI network for all the 34 genes with Cytoscape (settings: *Homo sapiens* and confidence > 0.4) and 10 hub genes with plugcluster Cytohubba by maximal clique centrality (MCC) method were screened. To be more specific, the 34 targets were as follows: CYP2D6, KCNA1, HTR7, DRD3, ADRA2A, OPRK1, CHRM1, CHRNB2, ADRA1A, HTR1A, DRD4, HTR1D, HTR2A, SLC6A3, ADRA2C, SLC6A4, PTPN11, HTR2C, KCNA2, MAPT, DRD2, ADRA1B, SIGMAR1, PPARA, CNR2, HTR2B, HTR1B, ADRA2B, CHRNA4, HTR6, OPRD1, KCNA5, OPRM1, and PTGS1 (Fig. 3a). Among which, the top 10 hub genes were DRD4, HTR1A, DRD2, OPRD1, DRD3, OPRM1, OPRK1, HTR1B, ADRA2C, and ADRA2A (Fig. 3b, c). The PPI network was rebuilt with top 8 hub genes and related active compounds of YB, resulting in 23 core compounds, which may be active therapeutic compounds related with pain relief in YB (Fig. 4).

### GO enrichment and KEGG enrichment

**Fig. 5:**
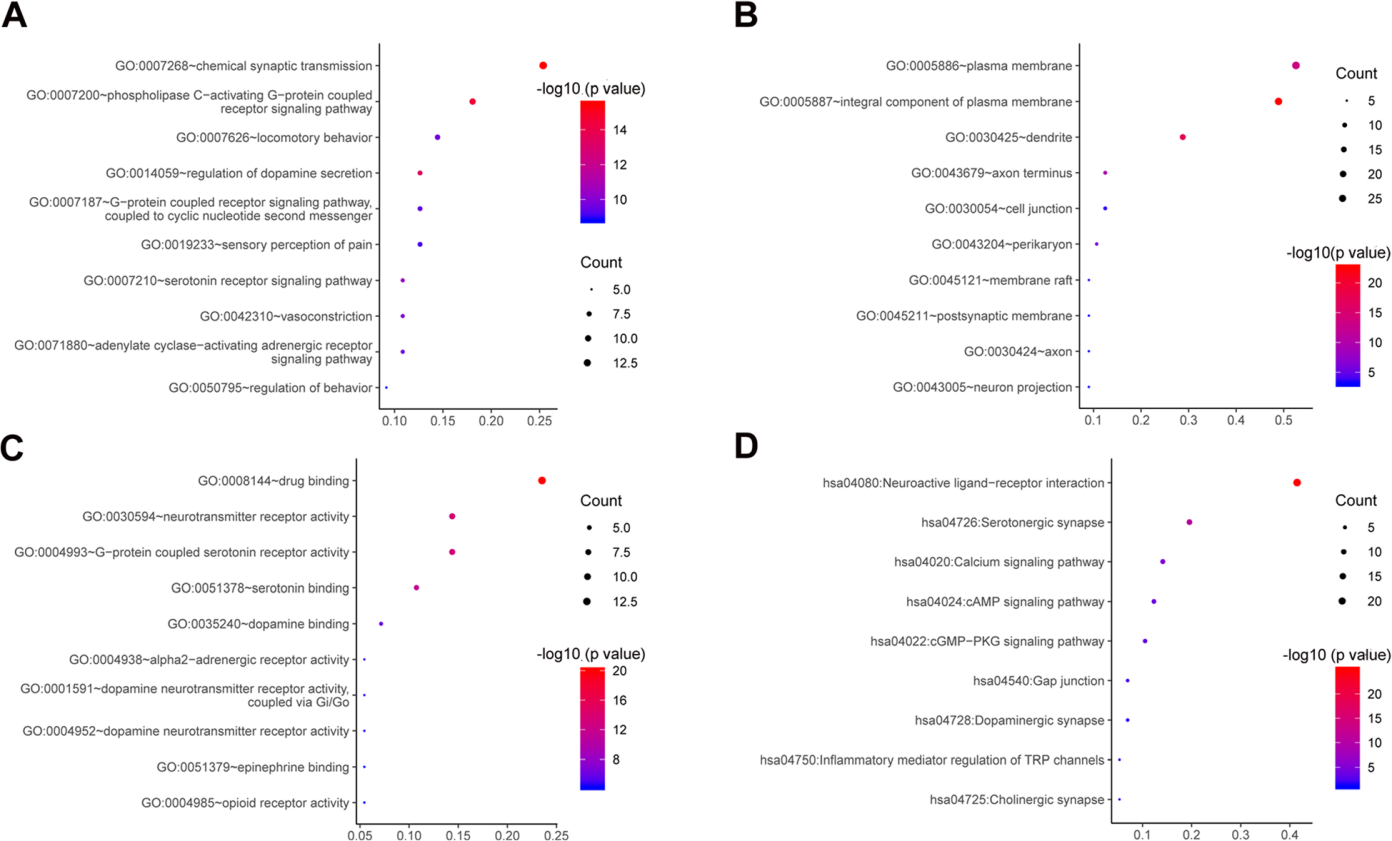
Gene enrichment (GO) analysis for the 34 shared YB compounds and pain-related target genes. The color represents the different -log10 (*p* values), while the size of the circle represents the counts. **a** Biological Process (BP); **b** Cellular component (CC); **c** Molecular Function (MF); **d** KEGG pathway analysis for the 34 shared YB compounds and pain-related target genes

To further explore the multiple mechanisms of YB as a therapy drug against pain, GO enrichment analysis of 34 target genes shared by YB and pain was performed using DAVID bioinformatics resources. The top 10 significantly enriched terms including biological process (BP), molecular function (MF), and cellular component (CC) are presented (*p*-value < 0.05) in Fig. 5a-c. The top 10 MF pathways included: drug binding, neurotransmitter receptor activity, G-protein coupled serotonin receptor activity, serotonin binding, dopamine binding, alpha2-adrenergic receptor activity, dopamine neurotransmitter receptor activity, dopamine neurotransmitter receptor activity, epinephrine binding, opioid receptor activity, which are all classical pathways involved in pain inducing and relieving. BP and CC terms analysis indicate that YB are mainly involved in chemical synaptic transmission, G protein coupled receptor signaling, and locomotory behavior. What’s more, YB are strongly related to the integral component of plasma membrane, cell junction, and dendrite.

The KEGG enrichment analysis of 34 target genes was performed to explore the potential biological pathways. We obtained 9 pathways in total which belong to several categories, including neuroactive ligand receptor interaction, serotonergic synapse, calcium signaling pathway, cAMP signaling pathway, dopaminergic synapse, etc. (Fig. 5d).

## Discussion

As Traditional Chinese Medicine (TCM) has been widely accepted around the world, there are still several problems to be addressed, among which, the active components and target genes have always been the issue and key point for TCM modernization [17, 18]. While existing methods mainly concerned the indicative ingredients and their potential pharmacological effects, network pharmacology study emerged as a more powerful method to identify active compounds and target genes due to multi-component and multi-target mode of TCM [19]. Le et al. reported that 7 alkaloids from Yuanhu and 8 coumarins from Baizhi were selected as active compounds by LC–MS/MS method [5]. In the present study, three databases were mined, resulting in 47 compounds in Yuanhu and 84 compounds in Baizhi. In total, 131 compounds were obtained from YB, which provided more compounds for further analysis. Normally, OB ≥ 30% and DL ≥ 0.18 are considered chemically suitable for drug development, they are used as the included criteria of bioactive compounds in most literatures [20]. In the current study, we calculated drug likeness probability and F (30% Bioavailability) probability of active compounds of YB using ADMETlab. Bioavailability, which is referred to as the degree and rate at which the active compound is absorbed by the systemic circulation, is calculated by Random forests (RF) method with accuracy of 0.669 and AUC score of 0.715 by fivefold cross validation. DL, which is defined as a complex balance of molecular properties and structure features, is closely related to bioactivity and bioavailability. DL is evaluated using one well-performed classification model with classification accuracy of 0.800 and AUC score of 0.867 by external test set [21]. An oral bioactivity of ≥20% is considered acceptable to identify compounds with accepted oral bioavailability. In this study, F ≥ 30% and DL ≥ 30% were used as screening criteria, which was stricter and more rigorous [22–24].

As shown in PPI network of Yuanhu, the most frequently targeted genes are ACHE, HTR1A, DRD2, HTR2A, HTR7, DRD1, HTR2B, DRD1, HTR7, HTR6, HTR5A, OPRM1, F3. Amongst them, the target genes in the neuro system accounted most, indicating that Yuanhu plays an important role in neuroprotection. While the top frequently targeted genes in Baizhi are CA12, CA9, CA7, PTGS1, DYRK1A, BACE1, BACE2, MBNL1. Among which, CAs, BACE, are involved in mechanism of neuronal protection against ischemia. PTGS1, known as Cyclooxygenase 1 (COX-1), are involved in osteogenic differentiation [25], cancer prevention and therapy [26], etc. And DYRK1A, MBNL1 are most involved in immune system [27], which suggest that Baizhi may be active in neuro-immune system. Dysfunctional pain is thought to arise from altered processing of nociceptive information in the central nervous system [28], and primary sensory neurons are involved in both acute and chronic pain [29], we deduct that YB may interact with neuro system to exert analgesia effect.

The PPI network also presents top 8 hub genes and its related 23 core compounds in YB. The top 8 genes actually belong to 3 families, dopamine receptors, 5-hydroxytryptamine (serotonin) receptors, and opioid receptors, which all are typical pain related genes, indicating that YB is definitely a pain-relieving candidate. Besides, the 34 genes targeted by YB contribute unevenly to the mechanism of analgesia effect. Take CYP2D6 for instance. CYP2D6 is the most frequently addressed candidate gene in the literature on pain, which is involved in the biological activation of codeine into morphine and tramadol into O-desmethyltramadol [30]. Based on the extensive evidence for this gene, the availability of guidelines for healthcare professionals, and the fact that active metabolites of codeine and tramadol are formed by this enzyme, this is a highly suitable biomarker for improving pain therapy in the clinic [31]. The sigma-1 receptor (SIGMAR1) is reported to be involved in pain modulation especially under pre-sensitized conditions [32], and related to CNS inflammation [33]. KCNA1 were found to be associated with membership in the mild pain class [34], endogenous Kcna2 antisense RNA was suggested as a therapeutic target for the treatment of neuropathic pain [35], while KCNA5 is mainly involved in drug uptake [36]. The alpha-2-adrenergic receptors targeted by YB, including ADRA2A, ADRA1A, ADRA2C, ADRA1B, ADRA2B, are involved in regulating the release of neurotransmitter molecules from sympathetic nerves and from adrenergic neurons in the central nervous system. SLC6A4, a member of neurotransmitter symporter family, can be targeted by psychomotor stimulants, such as amphetamines and cocaine and can terminate the action of serotonin and recycles it in a sodium-dependent manner. While SLC6A3 is a dopamine transporter. In addition, MAPT, CNR2 CHRM1, CHRNB2, CHRNA4 are all neuro-related proteins. While PTPN11, PPARA, PTGS1 have been shown to be more involved in cell proliferation. All the lines of evidence suggest that YB function as an analgesia through multi targets, amongst which, neuroprotection may account the most. This indicates that YB may be more suitable for chronic pain than acute ones, which is more related to inflammation.

The 23 compounds are as follows: Stylopine, Tetrahydropalmatine, Canadine, (+)-Corybulbine, (+)-Corydaline, Corydalmine, Corydine, Corypalmine, Glaucine, Isocorydine, Isocorypalmine, Lirioferine, (+)-N-Methyl Laurotetanine, Norisocorydine, Scoulerine, Tetrahydrocoptisine, Tetrahydrocorysamine, Thaliporphine, Yuanhunine, DL-Tetrahydropalmatine, codeine, thebaine. Among which, Glaucine, tetrahydropalmatine, canadine, corydaline, and tetrahydrocoptisine were determined relatively high in Rhizoma corydalis extract by HPLC-TOF/MS and they were absorbed into blood quickly [37]. Codeine is a well-known natural plant alkaloid commonly used to treat mild-to-moderate pain and cough [38]. Thebaine (paramorphine), chemically similar to codeine and morphine, is not used therapeutically but often converted to codeine for use. Dehydrocorybulbine (DHCB), has been identified as a dopamine receptor antagonist, exhibiting high to moderate binding affinities to sigma 1 and 2 receptors, serotonin 5-HT7 receptor, and histamine H2 receptors [39]. While (R)-glaucine appears to act as a positive allosteric modulator at the 5-HT2A receptor [40]. Levo-tetrahydropalmatine (L-THP) and Isocorypalmine exerted analgesic effects by agonism D1R and antagonism D2R [41, 42]. Levo-corydalmine attenuates vincristine-induced neuropathic pain by regulating the CXCL1/CXCR2 signaling pathway [43].

We also discovered several compounds in YB, which have not been reported to be related with analgesia in previous studies. However, they did have important functions in diverse diseases. For instance, tetrahydrocoptisine was reported to exert neuropsychopharmacological property in 1976 [44]. Thaliporphine, along with reperfusion therapy conferred cardioprotection via activation of opioid receptor [45]. Stylopine, which also exits in *Chelidonium majus* L. (Papaveraceae), is tested to have anti inflammation activity [46]. While Lirioferine and (+)-N-Methyl Laurotetanine were shown as good candidate with antileishmanial activity [47]. Isocorydine can selectively inhibit human cancer stem cells, which have an important role in the development of chemoresistance [48]. Scoulerine is a potent antimitotic compound and it merits further investigation as an anticancer drug [49]. These lines of evidence demonstrate that YB may act on multiple targets to play their pharmacological roles on pain. What’s more, little reports were found about Canadine, (+)-Corybulbine, (+)-Corydaline, Corydine, Corypalmine, Norisocorydine, Tetrahydrocorysamine, Yuanhunine, which may act as potential compounds for pain relief. It will be interesting to develop the potential active compounds as new anti-pain drugs.

## Conclusion

In summary, we found more potential compounds in YB involved in intrinsic control of pain besides the well-known ones, which still need further study to be verified about their specific roles in YB on pain. In addition, some crucial target genes, besides the already reported ones tested in animal experiments, such as CYP2D6, SIGMAR1, KCNA1, alpha-2-adrenergic receptors, SLC6A3, MAPT, CNR2 CHRM1, CHRNB2, CHRNA4, all may play significant roles in YB for attenuating pain.

## Supplementary information

**Additional file 1.**

**Additional file 2.**

## Data Availability

The datasets used and/or analyzed in the current study are available from the corresponding author on request.
